# Evaluating malaria programmes in moderate- and low-transmission settings: practical ways to generate robust evidence

**DOI:** 10.1186/s12936-020-03158-z

**Published:** 2020-02-18

**Authors:** Ruth A. Ashton, Debra Prosnitz, Andrew Andrada, Samantha Herrera, Yazoumé Yé

**Affiliations:** 1 MEASURE Evaluation, Center for Applied Malaria Research and Evaluation, Tulane School of Public Health and Tropical Medicine, Tulane University, 1440 Canal Street, Suite 2300, New Orleans, LA USA; 2 MEASURE Evaluation, ICF, Rockville, MD USA; 3 Save the Children, Washington, DC USA

**Keywords:** Process evaluation, Impact evaluation, Routine health information systems (RHIS), Surveillance, Monitoring evaluation, Decision-making, Malaria interventions, Low transmission, Moderate transmission

## Abstract

**Background:**

Many countries have made substantial progress in scaling-up and sustaining malaria intervention coverage, leading to more focalized and heterogeneous transmission in many settings. Evaluation provides valuable information for programmes to understand if interventions have been implemented as planned and with quality, if the programme had the intended impact on malaria burden, and to guide programmatic decision-making. Low-, moderate-, and heterogeneous-transmission settings present unique evaluation challenges because of dynamic and targeted intervention strategies. This paper provides illustration of evaluation approaches and methodologies for these transmission settings, and suggests how to answer evaluation questions specific to the local context.

**Methods:**

The Roll Back Malaria Monitoring and Evaluation Reference Group formed a task force in October 2017 to lead development of this framework. The task force includes representatives from National Malaria Programmes, funding agencies, and malaria research and implementing partners. The framework builds on existing guidance for process and outcome evaluations and impact evaluations specifically in high transmission settings.

**Results:**

The theory of change describes how evaluation questions asked by national malaria programmes in different contexts influence evaluation design. The transmission setting, existing stratification, and data quality and availability are also key considerations. The framework is intended for adaption by countries to their local context, and use for evaluation at sub-national level. Confirmed malaria incidence is recommended as the primary impact indicator due to its sensitivity to detect changes in low-transmission settings. It is expected that process evaluations provide sufficient evidence for programme monitoring and improvement, while impact evaluations are needed following adoption of new mixes of interventions, operational strategies, tools or policies, particularly in contexts of changing malaria epidemiology. Impact evaluations in low-, moderate-, or heterogeneous-transmission settings will likely use plausibility designs, and methods highlighted by the framework include interrupted time series, district-level dose–response analyses, and constructed control methods. Triangulating multiple data sources and analyses is important to strengthen the plausibility argument.

**Conclusions:**

This framework provides a structure to assist national malaria programmes and partners to design evaluations in low-, moderate- or heterogeneous-transmission settings. Emphasizing a continuous cycle along the causal pathway linking process evaluation to impact evaluation and then programmatic decision-making, the framework provides practical guidance in evaluation design, analysis, and interpretation to ensure that the evaluation meets national malaria programme priority questions and guides decision-making at national and sub-national levels.

## Background

Significant investments and subsequent scale-up of malaria interventions in the early 2000s led to decreased malaria transmission and a reduction in malaria cases in many countries [1, 2]. Transmission decreases in many countries has led to more focalized and heterogeneous malaria transmission, sub-nationally and among specific sub-populations. Concurrently, the landscape of interventions, operational strategies, and tools to measure prevalence and estimate transmission intensity has evolved.

Malaria programmes in low-, moderate-, and heterogeneous-settings present unique evaluation challenges. Many countries will implement multiple malaria interventions, and the effect of these interventions may differ between transmission zones. In some contexts, particularly those with heterogeneous-transmission, different intervention packages may be targeted to specific transmission risk zones, necessitating sub-national level data on population exposure to interventions over time to evaluate progress and impact. Refined evaluation methods should enable countries to measure the progress and impact of their malaria programmes even at low levels of transmission, in line with the Global Technical Strategy for Malaria (GTS) 2030 targets.

As countries go through epidemiological transition from high, through moderate to low malaria transmission, it becomes challenging to define the point at which an intervention has reached maturity to affect a health outcome. There is limited evidence to describe the intervention coverage level required to trigger declines in disease outcome; coverage targets are often arbitrary (e.g. 80%) or include the entire population, resulting in challenges for national malaria programmes (NMPs) in assessing programme performance and in knowing when it is appropriate to conduct impact evaluation. Ongoing improvement of routine health information systems (RHIS) such as the District Health Information System 2 (DHIS2) mean that routine surveillance data are increasingly available and complete at district level or lower, enabling evaluation at sub-national levels and permitting continuous analysis of trends over time. However, representativeness of RHIS data must be contextualized with an understanding of population access to and utilization of health services.

Since the 2000s, there have been efforts to design and implement appropriate methodologies to assess the impact of malaria intervention scale-up in high transmission settings, often by tracking changes in all-cause child mortality (ACCM) [3, 4]. These approaches have been used successfully in several malaria-endemic countries [5–8]. However, different evaluation approaches are needed in countries where transmission is low or heterogeneous.

This manuscript describes key concepts and examples from a framework for evaluation in low-, moderate-, or heterogeneous-transmission settings, developed by a task force of the Roll Back Malaria Monitoring and Evaluation Reference Group (RBM MERG) [9]. It highlights: (1) the increasing importance of quality routine surveillance data for evaluation, facilitating use of confirmed malaria incidence as an impact measure in low- and moderate-settings; (2) refined methods for impact evaluation in low- and moderate-transmission settings; and, (3) considerations for triangulating process and impact evaluation findings to lead to evidence-based decision making. The target audience for this includes monitoring and evaluation teams within NMPs, and implementing partners planning to support or conduct evaluations in low-, moderate-, and heterogeneous-transmission settings.

## Methods

The RBM MERG formed a task force in October 2017 to lead development of this framework. The task force includes representatives from NMPs, funding agencies, and malaria research and implementing partners (contributors named in Acknowledgements). The scope and objectives of the framework were informed through a review and synthesis of existing documents and tools for malaria surveillance, monitoring, and evaluation and through RBM MERG and task force discussion. The framework builds on existing guidance for process and outcome evaluations and impact evaluations specifically in high transmission settings [3, 4, 10, 11].

Briefly, the synthesis of existing tools and documents was completed October 2017, followed by a launch meeting in February 2018 in Washington DC where 25 taskforce members discussed findings of the review and agreed on the scope and aims of the framework. An event at the Multilateral Initiative on Malaria conference in April 2018 solicited additional input on the framework scope from country NMP teams. A full draft of the framework was circulated to the task force in June and again in August 2018 for feedback. A working session was held with task force members in September 2018 at the 29th MERG meeting to consolidate feedback and modifications to the framework. An external review of the framework was conducted by three malaria and/or evaluation experts in late 2018, and the final framework launched in April 2019.

Membership of the RBM MERG evaluation taskforce is voluntary. Development of this framework was through a consultative process and did not use focus group discussions or any other formal research methodologies.

## Results

### General considerations for evaluation in low-, moderate- and heterogenous-transmission settings

**Fig. 1:**
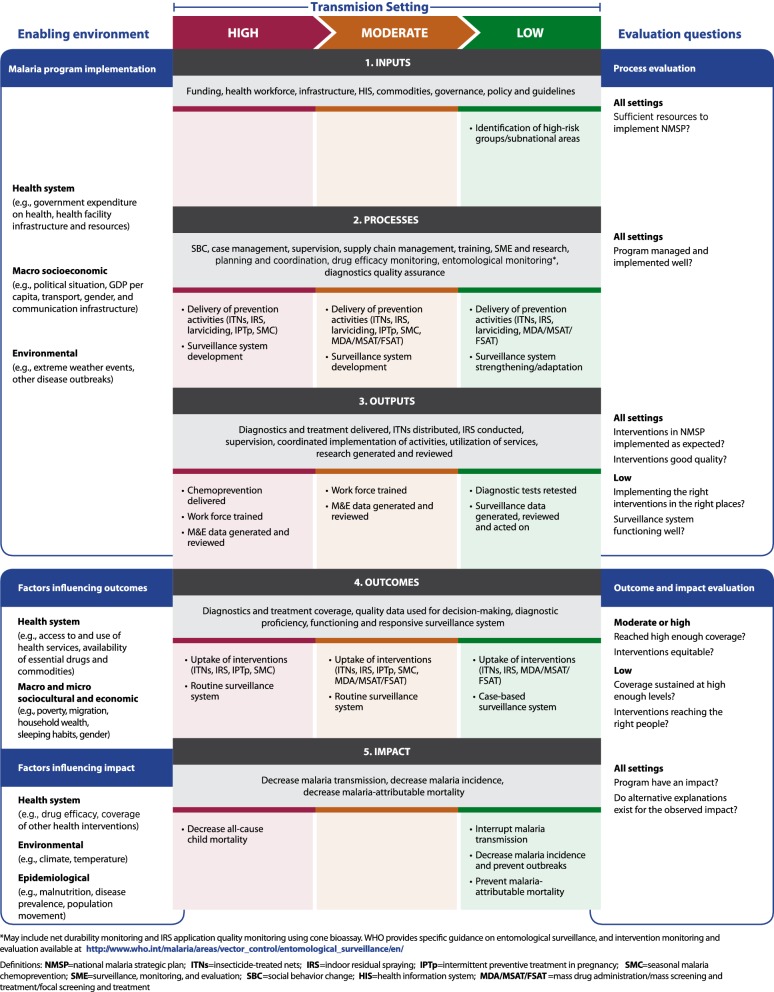
Theory of change for the framework to evaluate malaria programmes in low- and heterogeneous transmission settings. Transmission settings are defined according to WHO classifications: high [≥ 35% *Plasmodium falciparum* parasite rate (PfPR) or ≥ 450 cases per 1000 population annual parasite incidence (API)], moderate (10–35% PfPR or 250–450 API), and low (1–10% PfPR or 100–250 API). Elimination and very low transmission (< 1% PfPR but > 0% PfPR or < 100 API) are not considered in this framework

Capturing multiple and changing transmission settings, the framework theory of change (Fig. 1) emphasizes the need to use routine surveillance, monitoring and evaluation to (1) ensure quality and appropriateness of malaria control interventions; and, (2) shift focus to sub-national areas and sub-populations in which less progress has been made, or that have more focalized malaria transmission. The theory of change includes high- to low-transmission settings (as defined by WHO [12]), but this manuscript focusses on evaluation in moderate- [250–450 cases per 1000 annual parasite incidence (API)], low- (100–250 per 1000 API), and heterogeneous-transmission settings. Very-low transmission settings (< 100 per 1000 API) are excluded, since existing guidance is available for strategic planning and evidence generation in areas approaching elimination [13, 14].

**Fig. 2:**
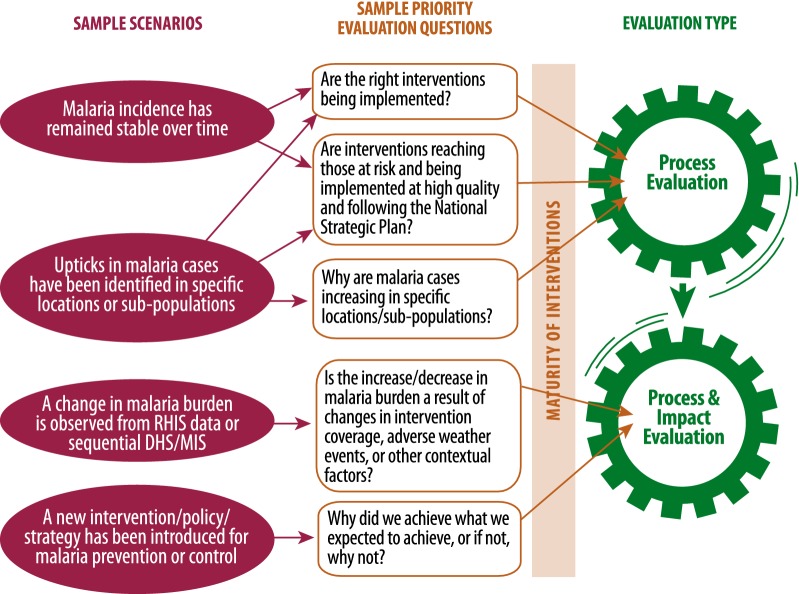
Example scenario diagram to assist in determining the type of evaluation that may answer priority evaluation questions

The design of an evaluation is influenced by priority evaluation questions, transmission setting, data sources, and quality of data available, as well as interventions applied, maturity of interventions, and strategies used to introduce or scale up these interventions. Figure 2 presents illustrative examples of evaluation questions that may be asked by NMPs, policy makers or partners. This scenario diagram illustrates that the combination of transmission setting and malaria intervention mix drive decisions of which type of evaluation to implement. In low-, moderate-, and heterogeneous-transmission settings, there is a need to ensure that intervention coverage and maturity have reached a level sufficient to trigger a decline in malaria incidence (impact indicator); only then an impact evaluation may be implemented. If intervention coverage and maturity are below optimum, the focus should be on process evaluation.

#### Linking process and impact evaluation

In low-transmission settings, it is expected that process evaluations provide sufficient evidence for programme improvement and course correction, tracking if interventions and policies are implemented at the targeted level and quality, and to identify any areas where outputs are not as expected. Impact evaluations are likely needed in these settings following adoption and scale-up of new mixes of interventions, operational strategies, tools, or policies, especially in contexts of changing malaria epidemiology. Consequently, process evaluation can both advise when impact evaluation may be useful (achieved intervention maturity, evaluation questions cannot be answered by process evaluation alone), and can explain results of an impact evaluation. Recommendations for optimal evaluation timing and planning are described further in the full frameworks [3, 9]. Linking process and impact evaluation of a NMP are critically important when examining a package of interventions, and answering questions such as: *Why did we achieve what we expected to achieve, and if not, why not?* Example scenarios, evaluation questions and suggested combination of process and impact evaluations are provided in Fig. 2.

#### Process-focussed questions

Questions such as “*Are interventions being implemented with good quality and following the national strategic plan?”* are common across transmission settings, and approaches for process evaluation are discussed in detail elsewhere [3, 9, 10]. By tying together programme inputs (e.g., policies, resources), the processes or interventions implemented, and the outputs of a programme (e.g., types, quantity and quality of services and interventions), to the achieved outcomes, process evaluations can characterize the strength or intensity of programme implementation. For evaluation questions such as *“Why are cases increasing in specific locations or among specific populations?”* and *“Are existing interventions still present at high coverage and still effective?”,* process evaluation is a part of, or precursor to impact evaluation, since inadequate implementation of a programme may lead to reduced impact.

#### Impact evaluation

Impact evaluation aims to determine whether the programme as a whole had an impact on malaria transmission and malaria-attributable morbidity and mortality, and assess the changes in impact measures that can be plausibly attributed to the particular package of interventions implemented by a NMP in the evaluation area. Questions such as: “*Is the increase or decrease in malaria burden a result of changes in intervention coverage, adverse weather events, or other contextual factors?”* can be answered by impact evaluation. Additional detail on use of quasi-experimental evaluation methodologies to develop plausibility arguments is provided in the full framework [9].

### Evaluation indicators and data sources in low-, moderate- and heterogenous-transmission settings

#### Confirmed case incidence as primary impact indicator

In low-, moderate-, or heterogeneous-transmission settings, where the number of malaria-attributable deaths is lower and surveillance systems are often more mature, the recommended primary indicator of impact is confirmed malaria case incidence. Confirmed malaria case incidence is collected routinely by most national health information systems, is available at sub-national levels, and is sensitive to short- and long-term changes. Confirmed incidence is limited by capturing only those malaria cases that present within the public health system and those that receive a confirmatory diagnostic test; health system contextual factors should be accounted for when interpreting results [15]. Secondary impact indicators and a comprehensive list of other evaluation indicators have been described elsewhere [3, 9] and are detailed in Additional file 1. ACCM has been recommended as a proxy indicator for assessing impact of malaria programmes in highly endemic settings due assessment of ACCM in national surveys and lack of available information on malaria-specific mortality [11, 12, 16, 17]. In heterogeneous-transmission settings or in contexts with limited routine data, it may be appropriate to use ACCM as the impact indicator in areas with moderate-transmission, and incidence in other areas.

#### Contextual information

In preparation for analysis and interpretation, potentially relevant contextual factors should be incorporated into a causal diagram or logic model to describe the hypothesized relationship between these factors and programme coverage and impact variables. Health system, environmental, epidemiological, sociocultural, economic, and other contextual factors can indirectly or directly influence programme implementation, malaria transmission, and malaria-related morbidity and mortality. The interplay between contextual factors and the transmission setting may be different, and how contextual factors influence outcomes and the impact of a programme may vary across transmission settings. If quantitative data for contextual factors (e.g. deforestation rate) are available they should be reviewed for quality and relevance for inclusion as a variable in evaluation analyses [18–20]. Qualitative contextual information such as anomalies (e.g., extreme weather events) [21], ecological changes (e.g., deforestation) [22], and information that are more anecdotal (e.g., disrupted delivery of interventions) [23] can be used to inform a plausibility argument [3]. Sources of contextual data and their relationship with malaria burden are discussed in detail elsewhere [9, 11].

#### Additional indicators and data sources for low-, moderate- and heterogenous-transmission settings

Population-based household surveys remain an important data source for national or sub-national estimates of reported insecticide-treated net (ITN) and intermittent preventive treatment in pregnancy (IPTp) coverage and access to health services. Surveys may also capture malaria infections external to the public health system through biomarker data (polymerase chain reaction (PCR) and serological analyses), and febrile illnesses reported by survey participants [24, 25]. In low-transmission settings, surveys increasingly include molecular methods such as PCR to detect low-density *Plasmodium* infections which may be missed by rapid diagnostic test (RDT) [26], or include serological methods which assess both recent and historical exposure to *Plasmodium* and can be used to estimate changes in transmission over time [27–29].

In Cambodia for example, successive national malaria surveys have included both PCR and detection of antibodies to *Plasmodium falciparum* and *Plasmodium vivax* in order to define areas of ongoing transmission, and to provide evidence to refine existing stratification and improve targeting of interventions [9]. The decision to collect seropositivity and PCR data was driven by a need to provide more granular information about where transmission was occurring than was possible using microscopy: surveys in 2010 and 2013 found *P. falciparum* prevalence by microscopy of 0.3 and 0.04%, respectively, but *P. falciparum* seropositivity was 11.5% in 2010 and 8.5% in 2013. Furthermore, the Cambodia data illustrates the utility of seroconversion rate (SCR, proportion of individuals becoming seropositive each year, analogous to force of infection) estimated from these survey data, with a decline SCR in adults across successive surveys indicating reduced exposure, but consistent increases in SCR around 14 years of age indicating occupational exposure (forest work). The use of PCR and serology data therefore provided additional evidence to inform intervention targeting of geographic areas and specific populations, complementing existing data on confirmed malaria incidence from RHIS. These additional tools and indicators continue to be refined and evaluated in different settings, and are particularly valuable for evaluation in moderate- and low-transmission settings [30].

### Refined evaluation design and methodologies

#### Incorporating epidemiological stratification in evaluation of heterogenous-transmission settings

Countries with low and heterogeneous malaria transmission often use a system of risk stratification to define areas of the country by their receptivity to malaria transmission. Consequently, malaria intervention packages, including surveillance, may differ by stratum. In Cambodia, the definition of risk strata has been refined over time (< 2 km or < 5 km from forested areas), creating additional complexity in impact evaluation. Evaluators took the approach of excluding 2004 survey data that had a more restrictive definition of risk zone, to enable comparison of equivalent areas over time (2007–2013). A stratified approach was used to describe cluster-level seroprevalence in each risk zone over time, revealing that exposure to *Plasmodium* became increasingly focused in the < 1 km from forest risk zone over time [9].

Strata should not be considered as simply geographical units; in settings where malaria risk is linked with demographic and behavioural factors, a stratified analysis among higher- and lower-risk populations may be appropriate. Considering strata within both process and impact evaluation is important to allow evaluators to identify sub-national locations where the programme performance or impact is lower than expected or lower than comparable locations. Understanding why these areas are not achieving the same performance and impact as other strata may require additional contextual data.

#### Addressing the lack of ‘control’ areas to assess impact

A key challenge of conducting impact evaluation of NMP activities is that it is often neither ethical nor feasible to introduce new interventions using a randomized approach, precluding the use of observed data from ‘control’ areas. In some settings, data from comparator areas that are otherwise similar to the implementation area aside from the intervention of interest were used to evaluate impact [31–33]. In Zanzibar, a pilot intervention of mass screening and treatment was evaluated by comparing data from intervention and control sites. In this case, control areas were selected to be similar to intervention in terms of transmission, location and population, but this approach to identification of controls is only applicable for early pilots of new interventions, not for routine application of interventions to all at-risk populations [32]. In Mali, routine implementation of seasonal malaria chemoprevention (SMC) was implemented in an intervention district and difference-in-difference regression analysis used to compare to a control district, determining that routine SMC reduced prevalence of malaria parasitaemia and anaemia. The control district did not receive SMC due to funding constraints, but was determined to be an appropriate comparison setting since it is adjacent to the intervention district, had similar geographic, social and demographic characteristics, and similar mix of other malaria interventions (both had ITN mass distribution, neither had indoor residual spray (IRS)) [31].

Quasi-experimental evaluation methods allow the evaluator to measure, to some extent, the causal link between the programme or intervention and the expected impact [34], an approach which is particularly valuable where randomization is not feasible, multiple health and development programmes are operating, and true contemporaneous control groups are not available [35, 36]. The potential applications of quasi-experimental designs and types of inference (probability, plausibility, adequacy) for evaluation of health interventions have been described elsewhere [34, 37, 38]. The use of a plausibility approach for impact evaluation for malaria programmes has been discussed in detail elsewhere [3].

#### Interrupted time series analyses

Interrupted time series (ITS) analyses are a robust approach to impact evaluation when a policy change or other intervention was introduced on a known date (Table 1) [39, 40]. ITS involves comparison of the level and mean trend in outcome indicators before and after a ‘breakpoint’ [41]. In Zanzibar, routinely reported surveillance data from public health facilities were used to estimate the confirmed malaria incidence each month from 2000 to the end of 2015 by *shehia* (administrative unit) [42]. Artemisinin-based combination therapy (ACT) was introduced in late 2003, and a combination of IRS and mass long-lasting ITN distribution began in 2006. An interrupted time series approach was chosen for the impact evaluation due to availability of incidence data both before and after the intervention packages were introduced, and since both ACT and vector control were introduced at known dates and with rapid roll-out (due to the relatively small geographical area). A segmented regression model was fit for three periods: (1) prior to introduction of ACT as first-line treatment; (2) during the period of ACT availability, but prior to vector control scale-up; and, (3) during the period of ACT availability, mass distribution of ITNs, and implementation of IRS. While this ITS model split into three ‘segments’, increasing the number of breakpoints in ITS analyses increases the probability of observed changes in the outcome being attributed to confounding factors [43]. Thus, the way in which the time series is divided into segments must balance information about how and when policy and intervention changes occurred, as well as other contextual changes. The strength of the ITS approach is its ability to account for secular trends, and to directly incorporate data on potential confounders. In Zanzibar, the model included a range of covariates to attempt to account for other causes of changing malaria incidence: number of facilities reporting data (reporting completeness), total all-cause facility attendance (access to health services), number of malaria tests performed (access to diagnostics), and anomalies in rainfall and temperature. By including potential confounding factors in the ITS model, the observed reductions in malaria incidence in Zanzibar can be plausibly attributed to the introduction of ACT and expansion of vector control interventions.

Multiple adaptations to ITS are possible, such as accounting for roll-out periods by incorporating lags between intervention and effect on outcome, and performing ITS on data from areas that received the programme and in equivalent comparison areas [44]. For ITS approaches, after fitting the ITS model it is possible to estimate a counterfactual by predicting the impact indicator assuming a continuation of baseline level and trend (and other covariate data) during the intervention period [42]. For example, ITS analyses were used to investigate the impact of stopping IRS on test positivity rates at two health facilities in Uganda [45]. An ITS approach was chosen since surveillance data were available covering the period before IRS, during sustained implementation, and after withdrawal of IRS. In Myanmar, an ITS analysis used an alternative impact indicator, exploring if an expansion of services provided by community health workers (CHWs) was associated with changes in blood examination rates by CHWs [46]. In the Myanmar study, the authors present similar findings from a range of alternative models to strengthen their conclusions (triangulation). In Uganda, ITS analysis was used to investigate if the introduction of community case management of malaria was associated with changes in health facility attendance in Uganda [47]. The authors used a simulated counterfactual (projecting the pre-intervention trend forward) to estimate the change in impact indicator at intervals throughout the intervention period.

#### Dose–response studies

Dose–response studies, also termed ‘national-evaluation platform approach’, make use of impact indicators available at sub-national level and at varying intensity of intervention or programme to examine the dose–response relationship between the intervention and impact indicator [35, 48, 49]. This approach is suitable in settings where data are available (or can be modelled) to describe both programme intensity and impact indicators at high spatial resolution (e.g., district level) and temporal resolution (e.g., monthly) (Table 1). In Zambia, a combination of district-level surveillance data and population survey data were used to evaluate the association between ITN coverage (intervention intensity, defined as number of ITNs per household at the district level, per year) and monthly malaria incidence [50]. Survey estimates of ITN coverage were not available for every year, so a geostatistical model was used to estimate longitudinal ITN coverage by month and district over the study period, by incorporating available district-level coverage estimates from surveys and ITN distribution data in a spatial framework. A regression model was used to assess the association between ITN coverage per district and confirmed malaria incidence including covariates to describe district reporting and testing rates, treatment seeking, health care access, climate (rainfall, temperature, vegetation index), calendar month and year. The model was fit in a Bayesian framework to account for spatial and temporal correlation. The analysis was stratified by low and high burden provinces to explore if the effect of ITN coverage on incidence differed between these settings. The results showed that while increasing ITN coverage by one net per household was associated with a 41% reduction in case incidence in areas of lower malaria burden, there was no association of ITN coverage with incidence in high burden provinces.

Dose–response models can also be used to estimate various alternative scenarios or counterfactuals, by simply fitting the model and predicting the outcome using appropriate intervention or programme coverage for the counterfactual scenario. In Zambia, this approach was used to estimate the ACCM rate that would have been expected in the absence of a top-up ITN distribution [51], using district-level models incorporating malaria intervention coverage, socio-economic indicators and other key health programme coverage estimates (immunization, antenatal care attendance, etc.), along with annual ACCM estimates. The analysis enabled generation of different scenarios of changing intervention coverage and ACCM, but also identified challenges in attribution of changes in the outcome indicator to specific interventions when multiple interventions are being scaled-up concurrently.

Methods such as regression discontinuity and propensity score matching enable statistical construction of controls from existing observational data (Table 1) [52]. The propensity score is a statistical matching approach, where a regression model is used to estimate individuals’ propensity to be exposed to the intervention, then exposed and unexposed individuals are matched based on their propensity scores. Propensity score matching uses survey data, and is therefore a useful method in settings without adequate routine data available and where only post-intervention data are available. Propensity score methods work best with large sample sizes, when the intervention is common but the outcome is rare, and evaluators can assume that no further unmeasured confounding variables exist that predict the propensity of receiving the intervention or are strongly correlated with the outcome of interest [43]. Propensity score matching has been used in evaluations of behaviour change messaging for ITN use [52] and impact of SMC [53], and a multi-country evaluation of the contribution of the President’s Malaria Initiative (PMI) to malaria burden reductions in sub-Saharan Africa [54], while few examples exist of use of regression discontinuity in malaria impact evaluations, despite its potential value [55]. In the example of propensity score matching to evaluate behaviour change messaging impact on ITN use, propensity scores were used because individual preferences often determine whether individuals participate in behaviour change communication (BCC) activities or receive BCC messages through various channels In the BCC study, the propensity scoring approach was complemented by a second analysis; both methods found a statistically significant effect of exposure to BCC messages on ITN use, strengthening the plausibility argument.

### Practical guidance for evaluation in low-, moderate-, and heterogenous-transmission settings

#### Linking process, outcome, and impact evaluation

It is essential to fully interpret findings and generate a national-level summary of results for non-specialist audiences, particularly in low- and heterogeneous-transmission settings. Including process evaluation findings in impact evaluation strengthens interpretation of impact evaluation results by understanding the ‘why’ behind the findings and thus enabling the programme to make necessary adjustments. Triangulation, the inclusion of multiple data sources and multiple analyses (e.g., comparing analyses using cross-sectional surveys and routine data), can improve the plausibility of findings using quasi-experimental evaluation approaches [35].

As an illustrative example linking process and impact evaluations, and data triangulation, consider an impact evaluation that found no change in malaria incidence after completing IRS in targeted districts, compared to unsprayed areas. Programmatic information about the IRS campaign and supporting entomological data are crucial to understanding why no impact was observed. Entomological sentinel site data might indicate the insecticide is no longer effective against local *Anopheles* species, while monitoring and supervision data from the IRS campaign might indicate operational problems, such as low coverage or non-adherence to spray protocols.

#### Changes in reporting methods and denominators

Changes in reporting methods and denominators are common challenges for evaluators, particularly when using routine data and evolving programme activities. In a setting where introduction of RDTs resulted in increased access to confirmatory diagnosis, a failure to account for this change could bias impact estimates downward, because increases in confirmed case incidence resulting from increased access to testing would be interpreted as a true increase in incidence. Including variables that capture changes in access to malaria diagnostics directly in analysis models (e.g., number tested, or proportion of all outpatient visits tested for malaria) can minimize potential bias from changing diagnostic test use. Since RHIS variables of ‘suspected malaria’ are interpreted inconsistently, being used to report either individuals who were treated for malaria when no diagnostics were available or any individual who was eligible for testing, caution should be used if including this variable in models as part of adjustments for changing diagnostic practices.

Another common scenario encountered by evaluators involves the routine malaria case count indicator definition changing from inclusion of presumptive and confirmed malaria to confirmed malaria only, and failure to account for this change could bias impact estimates upward as apparent decreases in malaria incidence would be attributed to the programme or intervention under evaluation. Presenting these data graphically with the time the indicator definition changed is a useful first step, and in settings with stable test positivity rates, estimates of confirmed malaria can be generated. A more cautious approach is to present analysis separately for each indicator definition period, or to complement analysis of routine data with data from relevant cross-sectional survey data.

Changes in survey sampling frames, changes in geographical boundaries, or re-stratification which results in a change in estimated population at risk over the evaluation period can also bias impact estimates either up or down. Evaluators should ensure they access the raw data to enable comparisons that use the same denominator definition over time. For example, if one region is no longer considered at risk of malaria, generating estimates of annual population incidence both including and excluding this region over the evaluation period can assist in explaining changes over time and where these changes occurred.

#### Challenge of endogeneity due to increasingly targeted approach of control programme activities

Endogeneity occurs where decisions are determined (in part or in entirety) by the impact indicator about which locations or populations receive an intervention. For example, IRS may be conducted in administrative units which had the highest incidence during the previous year. Failure to account for endogeneity can lead to erroneous evaluation conclusions that the intervention or programme was associated with increased malaria incidence. Analysis approaches such as regression discontinuity methods and use of instrumental variables may be useful in these contexts [55–57].

## Discussion

This framework builds on existing impact evaluation work by MERG in high transmission settings, expanding it to address settings along the continuum of malaria transmission, but with a particular focus on evaluation in moderate-, low-, and heterogeneous-transmission settings. This paper highlights examples from the field that illustrate solutions to common evaluation challenges in these settings and emphasize the importance of linking process to impact evaluation: linking implementation process to implementation strength, to then demonstrate programme impact on malaria morbidity. Major developments proposed include use of malaria incidence as the primary impact indicator for malaria evaluations in place of ACCM, and the likelihood that continuous process evaluation may be sufficient for many programmes to monitor progress of mature programmes, complemented by impact evaluations when new strategies, policies, or interventions are introduced.

Evaluation in low and heterogeneous malaria transmission settings can be complex with a mosaic approach of targeted interventions. As such, evaluation requires a more nuanced approach than previous efforts, which sought to simply demonstrate the impact of universal scale up of proven malaria interventions. While ACCM continues to be a relevant indicator for use in high-transmission settings, it is insufficiently sensitive in lower-transmission environments where few malaria-attributable deaths occur, particularly where adequate quality routine surveillance data enables the use of confirmed malaria incidence, a considerably more specific indicator.

Use of routine surveillance data in impact evaluation has been limited to date, due to concerns over data quality and potential for bias. However, following greatly increased access to confirmatory malaria diagnosis through introduction of RDTs, surveillance data offers the ability to use confirmed malaria incidence as an impact indicator in evaluations. Consideration of data quality is an important component of evaluation, since poor-quality data may result in misleading or incorrect evaluation findings. However, RHIS data do not need to be perfect to be used in evaluation, just of ‘adequate quality’. While a strict definition of adequate quality is impractical, problems with incomplete or missing data, creation of new health facilities, and increasing access to confirmatory diagnosis can be accounted for in data analysis [15], and formal data quality assessments can also be used to provide further information about RHIS data quality [58–61]. Furthermore, close investigation of data quality and identification of limitations in available data will guide interpretation of evaluation results, including reporting the direction of any potential bias that could result from the specific limitations of the RHIS data.

**Table 1:** A summary of impact evaluation study designs and methodologies

Methodology/study design	When is it useful?	What types of data can be used?	How robust is the design?
Interrupted time series	Policy change or other intervention introduced on a known date. Useful when no underlying contemporaneous control group, but can be adapted to include a control group	Time-series data (retrospective or prospective), ideally RHIS	Good. Considers secular trends and confounding factors, counterfactual can be estimated
Dose–response	When no clear intervention and comparison areas, but intervention at varying levels of intensity by district	Sub-national data (e.g., district-level) describing intervention, impact indicator, and potential confounders. Ideally RHIS. Requires data on process and activities to define ‘intensity’	Moderate, if high spatial and temporal resolution and confounders included. Can estimate counterfactuals for alternative programme coverage levels. Prone to confounding because intensity of intervention or program applied may be related to impact outcome
Constructed controls (matching or discontinuity designs, instrumental variables)	When no clear intervention and comparison areas, but differences in individual use and access to interventions, or eligibility criteria determine whether an individual or area received interventions. Useful for inference at the individual level	Individual-level data from cross-sectional survey data with large sample size, and all possible confounders measured	Moderate. Limited by availability of data from which to estimate controls. Often uses data from a single cross-sectional survey, and evaluation may have low power to identify changes where cross-sectional RDT positivity is the primary impact indicator
Stepped-wedge	Phased introduction of programme with or without randomization	RHIS or repeat cross-sectional surveys	Moderate. Important to account for other programmes or contextual changes occurring during the phased roll-out of program being evaluated

While impact evaluation has typically been largely driven by funding partners’ interests in demonstrating the impact of funding provided, this framework proposes an increased focus on evaluation of process, outcome and impact of NMPs, and engagement with stakeholders at all stages to ensure that the evaluation meets NMP priority questions and provides adequate feedback for ongoing improvement and adaptive management. In many low-transmission settings where the programme has already achieved scale-up of key interventions, evaluation activities are likely to primarily take the form of continuous process evaluation, being complemented by impact evaluation when a substantial change in policy, intervention, or strategy has taken place.

Persistent challenge of evaluations in low-, moderate-, or heterogeneous-transmission settings limits the application of this framework. These challenges, which are also priority areas for further operations research, include: benchmarking adequate quality RHIS data for use in evaluation; defining intervention maturity of malaria programmes and setting thresholds for implementation strength; fully accounting for endogeneity; and determining at what level of programme coverage measurable impact is expected.

## Conclusions

Well-designed and timely evaluation is important to strengthen malaria programmes and support continued progress in controlling malaria. This paper builds on existing guidance for evaluation in high-transmission settings by presenting a comprehensive approach to evaluation across the continuum of malaria transmission. Specifically, the manuscript highlights the importance of routine surveillance data for evaluation and the use of confirmed malaria incidence to measure impact in low-, moderate-, and heterogeneous-transmission settings. Underlining the linkage between process, output, outcome and impact evaluation, triangulation of findings is critical for guiding programmatic decision-making at national and sub-national levels. This manuscript presented refined methods for impact evaluation in low-, moderate-, and heterogeneous-transmission settings, with examples of their application to aid countries in adapting this framework to their local context.

## Key terms and definitions

*Process evaluation*: Method of assessing how a programme is being implemented; focusses on the programme’s operations, quality and coverage of implementation and service delivery.

*Impact evaluation*: Method of assessing the changes in an outcome that can be plausibly attributed to a particular intervention or package of interventions, such as a project, programme, or policy; seeks to answer cause-and-effect questions.

*Contribution*: Indicates that the exposure (to the programme/policy/intervention package) contributed to the observed change in impact indicator, however, additional factors (either unmeasured factors, or not included in impact evaluation analysis) may also have partly contributed to the change in impact indicator.

*Attribution*: Indicates evidence for a causal link between exposure (to the programme/policy/intervention package) and the impact indicator: the measured impact is attributable to the exposure.

*Counterfactual*: The state of affairs that would have happened in the absence of the exposure (e.g., malaria incidence that would have observed if the intervention was not in place).

*Implementation strength*: A quantitative measure of the level of quality and extent or scale of inputs to the implementation of a programme. Inputs should be all those in the programme’s design framework (e.g., logic model) including policies, strategies, and interventions *actually delivered* (Adapted from [62]).

*Intervention maturity*: Duration of intervention implementation *vis*-*à*-*vis* previously demonstrated ability to have the intended effect. For example, whether sufficient time has elapsed since ITN distribution for nets to have been hung in households and a consequent reduction in number of infectious mosquito bites to have occurred.

## Supplementary information

**Additional file 1: Table S1.** Monitoring and evaluation core indicator reference guide.

## Data Availability

Data sharing is not applicable to this article as no datasets were generated or analyzed during the current study.

## Abbreviations

ACCM: All-cause child mortality
ACT: Artemisinin-based combination therapy
API: Annual parasite incidence
BCC: Behaviour change communication
CHW: Community health worker
DHIS2: District Health Information System 2
GTS: Global Technical Strategy for Malaria
IPTp: Intermittent preventive treatment in pregnancy
IRS: Indoor residual spray
ITN: Insecticide-treated net
ITS: Interrupted time series
NMP: National malaria programme
PCR: Polymerase chain reaction
RBM MERG: Roll Back Malaria Monitoring and Evaluation Reference Group
RDT: Rapid diagnostic test
RHIS: Routine health information systems
SCR: Seroconversion rate
SMC: Seasonal malaria chemoprevention
WHO: World Health Organization
