# Supplementary material for: Evaluating malaria programmes in moderate- and low-transmission settings: practical ways to generate robust evidence
Source: Malar J. 2020 Feb 18;19:75. doi: 10.1186/s12936-020-03158-z (PMC7027277; doi:10.1186/s12936-020-03158-z)
Supplement: Supplementary file 1 — Additional file 1: Table S1. Monitoring and evaluation core indicator reference guide. [file 12936_2020_3158_MOESM1_ESM.docx]

# Additional File

Table S1. Monitoring and evaluation core indicator reference guide

|  | **Indicator** | **Numerator** | **Denominator** | **Source** | **Disaggregation** | **Comments** |
| --- | --- | --- | --- | --- | --- | --- |
| **Input** | |  |  |  |  |  |
|  | Expenditure per capita for malaria control or elimination [9] | Malaria expenditure (domestic and international) | Population at risk of malaria | Routine administrative and financial systems | Source of funds (e.g., domestic, private sector, household, international), program area, geographical area | Direct malaria expenditure can be reported if expenditures shared with other programs cannot be readily apportioned to malaria |
|  | Human resources: Number of health workers per 10,000 population [22] | Number of health workers X 10,000 | Population size | Census, routine administrative systems | Geographical area |  |
|  | Annual number of malaria commodities procured by type | Number of malaria commodities procured during one year |  | Routine program reporting | Type of commodity |  |
| **Output** | |  |  |  |  |  |
| **Social and behavior change communication** | | | | | | |
|  | Number and proportion of population at risk who recall hearing or seeing malaria messages within the past six months | Number of people at risk of malaria who recall hearing or seeing any malaria message during the last six months | Total number of survey respondents | Population-based household survey | Geographical area, age, sex |  |
| **Vector control** | | | | | | |
|  | Number of insecticide-treated nets (ITNs) distributed | Number of ITNs distributed during one year |  | Routine program reporting | Geographical area |  |
|  | Number and proportion of households targeted for indoor residual spraying (IRS) that received IRS | Number of households targeted for IRS that received IRS during the reporting period | Total number of households targeted for IRS during the reporting period | Routine program reporting | Geographical area |  |
|  | Number of areas targeted for larviciding that are covered | Number of areas targeted for larviciding that are covered during the reporting period |  | Routine program reporting | Geographical area |  |
|  | Number of entomological monitoring sites | Number of entomological monitoring sites |  | Routine program reporting |  |  |
| **Chemoprevention** | | | | | | |
|  | Number of sulfadoxine-pyrimethamine doses delivered for IPTp | Number of sulfadoxine-pyrimethamine doses delivered during the reporting period |  | Logistics management information system | Geographical area |  |
|  | Number of children ages 3–59 months who received the full number of courses of seasonal malaria chemoprevention (SMC) per transmission season [9] | Number of children ages 3–59 months who received the full number of courses of SMC in a transmission season |  | Routine health information system, census | Geographical area, type of facility, sex |  |
| **Diagnostic reporting** | | | | | | |
|  | Number and proportion of health facilities with microscopy or rapid diagnostic test (RDT) capability | Number of health facilities with microscopy or RDT capability | Total number of health facilities | Routine administrative reporting | Geographical area, type of facility |  |
|  | Number of blood slides taken and read | Number of blood slides taken and read |  | Routine health information system | Geographical area, type of facility |  |
|  | Number of RDTs done and read | Number of RDTs done and read |  | Routine health information system | Geographical area, type of facility |  |
|  | Number of microscopy slides cross-checked by national reference laboratory | Number of microscopy slides cross-checked by national reference laboratory |  | Routine health information system, routine program reporting | Geographical area, type of facility |  |
| **Treatment** | | | | | | |
|  | Number of first-line antimalarial treatment courses administered | Number of first-line antimalarial treatment courses administered during the reporting period |  | Logistics management information system | Age, sex |  |
|  | Number of pre-referral treatment courses administered | Number of pre-referral treatment courses administered during the reporting period |  | Logistics management information system | Age, sex |  |
|  | Number of radical cure treatment courses (primaquine or tafenoquine) administered (*P. vivax* settings) | Number of radical cure treatment course (primaquine or tafenoquine) administered during the reporting period |  | Logistics management information system | Age, sex |  |
|  | Number of single, low-dose primaquine treatment courses administered for *P. falciparum* transmission blocking | Number of single, low-dose primaquine treatment courses administered |  | Logistics management information system | Age, sex |  |
|  | Number of severe malaria cases referred | Number of severe malaria cases referred |  | Logistics management information system | Age, sex |  |
|  | Number of antimalarial treatment courses for severe malaria cases administered | Number of antimalarial treatment courses for severe malaria cases administered during the reporting period |  | Logistics management information system | Age, sex |  |
| **Commodities** | | | | | | |
|  | Number of health facilities with stockouts of key commodities for diagnostic testing | Number of health facility reports received on time during the month |  | Logistics management information system, health facility survey | Geographical area, type of facility |  |
|  | Number of health facilities with stockouts of key malaria drugs | Number of health facility reports received that are complete during the month |  | Logistics management information system, health facility survey | Geographical area, type of facility |  |
| **Surveillance** | | | | | | |
|  | Number and proportion of expected health facilities reports received on time | Number of health facility reports received on time during the month | Number of health facilities | Routine health information system | Geographical area, type of facility |  |
|  | Number and proportion of expected health facility reports received that are complete | Number of health facility reports received that are complete during the month | Number of health facilities | Routine health information system, data quality audit | Geographical area, type of facility | May require a data quality audit to assess the completeness of forms |
| **Training and supervision** | | | | | | |
|  | Number and proportion of health facilities with a trained clinician in case management | Number of health facilities with a trained clinician in case management | Number of health facilities | Routine administrative reporting | Geographical area, type of facility |  |
|  | Number and proportion of health facilities with staff trained in surveillance, monitoring, and evaluation | Number of health facilities with staff trained in surveillance, monitoring, and evaluation | Number of health facilities | Routine administrative reporting | Geographical area, type of facility |  |
|  | Number and proportion of health facilities that received supervisory visits in the reporting period | Number of health facilities that received a supervisory visit during the reporting period | Number of health facilities | Routine program reporting | Geographical area, type of facility |  |
| **Drug and insecticide efficacy monitoring** | | | | | | |
|  | Number of drug efficacy studies completed | Number of drug efficacy studies completed during the reporting period |  | Routine program reporting | Geographical area |  |
|  | Number of insecticide efficacy studies completed | Number of insecticide efficacy studies completed during the reporting period |  | Routine program reporting | Geographical area |  |
| **Outcome** | |  |  |  |  |  |
| **Malaria knowledge** | | | | | | |
|  | Proportion of population at risk who know the main symptom of malaria | Number of people who know that the main sign/symptom of malaria is fever | Number of people surveyed | Population-based household survey | Geographical area, age, sex |  |
|  | Proportion of population at risk who know the treatment for malaria | Number of people who know that the appropriate treatment for malaria is artemisinin-based combination therapy | Number of people surveyed | Population-based household survey | Geographical area, age, sex |  |
|  | Proportion of population at risk who know preventive measures for malaria | Proportion of people who know that the primary preventive measures for malaria include using bed nets, taking preventive medication during pregnancy, taking seasonal prophylaxis, or having house sprayed with insecticide | Number of people surveyed | Population-based household survey | Geographical area, age, sex |  |
| **Vector control** | | | | | | |
|  | Proportion of population at risk with access to an ITN in their household | Total number of individuals at risk for malaria who could sleep under an ITN if each ITN in the household is used by two people | Total number of individuals at risk of malaria who spent the previous night in surveyed households | Population-based household survey | Geographical area, age, sex, urban or rural, wealth index, household size |  |
|  | Proportion of population at risk that slept under an ITN the previous night | Number of individuals sleeping under an ITN the previous night | Total number of individuals who spent the previous night in surveyed households | Population-based household survey | Geographical area, urban or rural, wealth index, educational status, pregnancy status, age, sex, household size |  |
|  | Proportion of population at risk protected by IRS during previous 12 months | Number of people protected by IRS in the previous 12 months | Population at risk of malaria | National malaria program records, census | Geographical area, age, sex |  |
|  | Proportion of population at risk with access to an ITN in their household | Number of people at risk with access to an ITN in their household | Population at risk of malaria | Routine program reporting | Geographical area, urban or rural, wealth index, household size |  |
|  | Proportion of adult female vectors alive after exposure to insecticide (resistance frequency) | Number of dead or incapacitated *Anopheles* malaria vector | Total number of *Anopheles* malaria vectors exposed to a discriminating concentration of insecticide in standard bioassays | Special study |  |  |
|  | Resistance to insecticide status | Number of *Anopheles* malaria vectors confirmed resistant, possibly resistant, or susceptible | Total number of *Anopheles* malaria vectors exposed to a discriminating concentration of insecticide in standard bioassays | Special study |  |  |
| **Chemoprevention** | | | | | | |
|  | Proportion of pregnant women who received three or more doses of intermittent preventive treatment in pregnancy | Number of women who received three or more doses of intermittent preventive treatment in pregnancy | Number of expected pregnancies (routine health information system)  Number of women ages 15–49 surveyed who had a live birth in the last two years (population-based household survey) | Routine health information system, population-based household survey | Geographical area, age |  |
|  | Proportion of eligible children ages 3–59 months who received the full number of courses of SMC per transmission season | Number of children ages 3–59 months who received the full number of courses of SMC in a transmission season | Number of children ages 3–59 months requiring SMC | Routine health information system, census | Geographical area, age, sex |  |
| **Diagnostic testing** | | | | | | |
|  | Proportion of patients tested among all febrile patients | Number of febrile patients tested for malaria | Number of all febrile patients | Routine health information system, population-based survey, health facility survey | Geographical area, type of facility, age, sex |  |
|  | Proportion of cases confirmed by a parasitological test of all reported cases | Number of cases confirmed by a parasitological test | Number of reported cases | Routine health information system, health facility survey | Geographical area, type of facility, age, sex |  |
|  | Proportion of health facilities without stockouts of key commodities for diagnostic testing | Number of health facility months with no stockouts of key commodities for diagnostic testing | Number of health facility months | Routine health information system, health facility survey | Geographical area, type of facility |  |
|  | Proportion of microscopy results cross-checked by national reference laboratory | Number of microscopy results cross-checked by national reference laboratory | Total number of microscopy results | Routine health information system, health facility survey | Geographical area, type of facility | Disaggregate by positive and negative results |
|  | Proportion of microscopists achieving both sensitivity and specificity greater than 90 percent during proficiency tests | Number of microscopists achieving both sensitivity and specificity greater than 90 percent during proficiency tests | Total number of microscopists assessed through proficiency tests |  | Geographical area, type of facility |  |
| **Treatment** | | | | | | |
|  | Proportion of children under five with fever in the past two weeks for whom advice or treatment was sought from a health provider | Number of children under five with fever in the past two weeks for whom advice or treatment was sought from a health provider | Total number of children under five with fever in the past two weeks | Population-based household survey | Geographical area, urban or rural, wealth index, educational status, sex |  |
|  | Proportion of patients with confirmed malaria who received first-line antimalarial treatment according to national policy [9] | Number of patients with confirmed malaria who received first-line antimalarial treatment according to national policy | Total number of confirmed malaria case (includes cases found both passive and active surveillance) | Routine health information system, health facility survey or audit | Geographical area, type of facility, parasite species, age, sex | Will likely be more accurate from health facility survey or audit, because routine data will likely presume cases were treated according to national policy |
|  | Proportion of patients with *P. vivax* or *P. ovale* infection who received radical cure treatment (primaquine or tafenoquine)[9] | Number of patients with a confirmed *P. vivax* or *P. ovale* infection who received radical cure treatment (primaquine or tafenoquine) | Number of patients with confirmed *P. vivax* or *P. ovale* infection | Routine health information system, health facility survey | Geographical area, type of facility, parasite species, age, sex |  |
|  | Proportion of confirmed *P. falciparum* cases who received single, low-dose primaquine | Number of confirmed *P. falciparum* cases who received single, low-dose primaquine | Total number of confirmed *P. falciparum* cases | Routine health information system, health facility survey | Geographical area, type of facility, age, sex |  |
|  | Proportion of severe malaria cases that were referred | Number of patients with severe malaria who were referred | Number of patients with severe malaria | Routine health information system, health facility survey | Geographical area, type of facility, age, sex | Denominator for this indicator may not be collected and the indicator may not be able to be calculated |
|  | Proportion of referred patients with severe malaria that received pre-referral treatment | Number of referred patients with severe malaria that received pre‑referral treatment | Number of patients with severe malaria that were referred | Routine health information system, health facility survey | Geographical area, type of facility, age, sex |  |
|  | Proportion of health facility months without stockouts of first-line treatments (includes treatment for severe anemia) | Number of health facility months without stockouts of first-line treatments | Number of health facility months | Routine health information system, health facility survey | Geographical area, type of facility | Disaggregated by type of treatment (malaria and anemia) |
|  | Proportion of patients with confirmed malaria with adequate clinical and parasitological response | Number of patients with confirmed malaria with adequate clinical and parasitological response on day 28 (or 42) | Number of patients with confirmed malaria that were treated according to national policy and assessed on day 28 (or 42) | Therapeutic efficacy study | Geographical area/sentinel site, age, sex |  |
| **Surveillance** | | | | | | |
|  | Proportion of malaria cases detected by surveillance systems | Number of confirmed malaria cases identified through active and passive surveillance over 1 year X 1,000 | Estimated number of malaria cases over 1 year X 1,000 | Routine health information system | Geographical area, age, sex | Estimated number of malaria cases (denominator) should include the proportion of patients who seek care, proportion who receive a diagnostic test, and proportion of health facility reports received |
|  | Annual blood examination rate | Number of patients receiving a parasitological test during one year | Mid-year number of people at risk for malaria | Routine health information system | Geographical area, type of facility |  |
|  | Proportion of expected health facility reports received | Number of reports received from health facilities during the reporting period | Number of reports expected from health facilities during the reporting period (number of health facilities multiplied by the number of reports expected per health facility during the reporting period) | Routine health information system | Geographical area, type of facility |  |
|  | Number and proportion of malaria epidemics detected within two weeks [23, 24] | Number of malaria epidemics detected within two weeks | Number of malaria epidemics detected | Routine health information system | Geographical area | Indicator should be measured within a one-year time frame |
|  | Number and proportion of suspected malaria outbreaks investigated | Number of malaria suspected outbreaks investigated | Total number of suspected malaria outbreaks |  |  |  |
|  | Number and proportion of malaria outbreaks responded to in a timely manner | Number of malaria outbreaks responded to in a timely manner | Total number of malaria outbreaks |  |  |  |
|  | Proportion of inpatient deaths due to malaria (e.g., case fatality rate) | Number of inpatient deaths due to malaria | Total number of inpatient deaths | Routine health information system, health and demographic surveillance system (HDSS)/sentinel sites | Geographical area, age, sex |  |
| **Impact** | | | | | | |
|  | Malaria case incidence: number and rate per 1,000 people per year (disaggregate by species and active and passive case detection for low-transmission settings) | Number of confirmed malaria cases identified by active and passive surveillance during 1 year X 1,000 | Mid-year number of people at risk for malaria infection during reporting year | Routine health information system, community health information system, HDSS/sentinel sites | Geographical area or focus, risk group, active versus passive case detection, age, sex, and species | May report number of cases when incidence is low |
|  | Malaria test positivity rate | Number of confirmed malaria cases | Number of patients who received a parasitological test | Routine health information system, community health information system, HDSS/sentinel sites | Geographical area, age, sex, parasite species |  |
|  | Proportion of admissions for malaria | Number of inpatient admissions for malaria | Total number of inpatient admissions | Routine health information system, HDSS/sentinel sites | Geographical area, age, sex |  |
|  | Malaria mortality: number and rate per 100,000 people per year | Number of malaria-specific deaths reported in the previous year X 10,000 | Mid-year number of people at risk for infection during the reporting year | Routine health information system, HDSS/sentinel sites, civil registration and vital statistics | Geographical area, age, sex, risk group and parasite species | May report number of deaths when mortality rate is low |
|  | All-cause child mortality (Number of deaths among children ages 0–59 months per 1,000 live births) | Number of deaths among ages children 0–59 months per 1,000 live births | 1,000 live births | Population-based household surveys, census, civil registration and vital statistics, HDSS/sentinel sites | Age |  |
|  | Annual number of malaria outbreaks reported | Number of malaria outbreaks reported in the previous year |  | Routine health information system, routine program reporting |  |  |
|  | Parasite prevalence: proportion of population with infection with malaria parasites | Number of people with malaria infection detected by RDT or microscopy | Total number of people tested for malaria parasites by RDT or microscopy | Population-based household survey, special study | Geographical area, urban or rural, wealth index, educational level, sex |  |
|  | Seroprevalence | Number of people who tested positive for antimalarial antibodies | Total number of people tested for antimalarial antibodies | Population-based household survey, special study | Geographical area, age, sex |  |

Source: Drawn from the World Health Organization Malaria Surveillance, Monitoring and Evaluation Reference Manual [9] and the World Health Organization Malaria Manual for Elimination Scenario Planning [10]
